# P-73. Unique Pathogen Distribution in Patients Undergoing Chronic Antibiotic Suppression for Cardiac Implantable Electronic Device Infection

**DOI:** 10.1093/ofid/ofaf695.302

**Published:** 2026-01-11

**Authors:** Shari J Zaslow, Daniel C DeSimone, Larry M Baddour, Supavit Chesdachai

**Affiliations:** Mayo Clinic, Rochester, MN; Mayo Clinic, Rochester, MN; Mayo Clinic College of Medicine, Rochester, MN; Mayo Clinic, Rochester, MN

## Abstract

**Background:**

Cardiac implantable electronic device infection (CIEDI) is a serious complication, occurring in approximately 1% of implantations. In addition to antimicrobial therapy, complete device extraction is considered essential for definitive cure. However, this may not always be feasible due to a variety of factors. In select cases, long-term chronic antibiotic suppression (CAS) has been used, yet clinical characteristics and outcomes of patients who received CAS have scarcely been reported.

**Methods:**

A descriptive study analyzing all adult (age > 18) patients with definite CIEDI who received CAS between 2016 and 2023 across the Mayo Clinic Enterprise (Arizona, Florida, Minnesota, and Upper Midwest) was conducted. Patients with either ventricular assist devices or fungal infection were excluded.

**Results:**

A total of 321 patients were diagnosed with definite CIEDI during the study period. A total of 37 (8.7%) patients received CAS due to device retention. The median age was 77.5 [IQR 70.5, 87.5] with 32.4% female. Permanent pacemakers were involved in most (64.9%) infections. Approximately one-third (32.4%) of patients had a prosthetic valve. The median time from initial device implantation to CIEDI was 10.6 years [IQR 4.8, 18.1]. CIED-related infective endocarditis was present in 70.3% of cases and 67.6% had lead involvement. The most commonly isolated organisms were *Staphylococcus aureus* (29.7%), coagulase-negative staphylococci (27%), *Enterococcus faecalis* (13.5%) and Streptococci (13.5%). Other pathogens or culture-negative cases comprised 16.3%. The primary reason for device retention and CAS was poor surgical candidacy. Doxycycline was the most commonly used agent for CAS, accounting for 37.8% of cases. The average length of suppression was 469.0 days [IQR158.0, 1264.0]. The rate of relapse was 8.1% with all occurring within 90 days of diagnosis. One year mortality was 27.0%.

**Conclusion:**

CAS may be a feasible option for patients with device retention, although a small proportion experience CIEDI relapse. Unexpectedly, the pathogen distribution among CAS recipients differed from that seen in a general CIED infection population, highlighting selection bias and the distinct clinical characteristics of patients managed with CAS.

**Disclosures:**

Larry M. Baddour, MD, UpToDate, Inc.: Royalty payments (authorship duties).

